# Cellular and molecular alterations to muscles and neuromuscular synapses in a mouse model of *MEGF10*-related myopathy

**DOI:** 10.1186/s13395-024-00342-6

**Published:** 2024-05-17

**Authors:** Devin Juros, Mary Flordelys Avila, Robert Louis Hastings, Ariane Pendragon, Liam Wilson, Jeremy Kay, Gregorio Valdez

**Affiliations:** 1 Department of Molecular Biology, Cell Biology and Biochemistry, Brown University, 70 Ship St, Providence, RI 02903 USA; 2 Pathobiology Graduate Program, Brown University, Providence, RI USA; 3 Department of Neurobiology, Duke University School of Medicine, Durham, NC USA; 4 Department of Ophthalmology, Duke University School of Medicine, Durham, NC USA; 5 Center for Translational Neuroscience, Robert J. and Nancy D. Carney Institute for Brain Science, Center on the Biology of Aging, Brown University, Providence, RI USA

**Keywords:** *Megf10*, Myopathy, Myogenesis, Neuromuscular junction, Perisynaptic Schwann cells

## Abstract

Loss-of-function mutations in *MEGF10* lead to a rare and understudied neuromuscular disorder known as *MEGF10*-related myopathy. There are no treatments for the progressive respiratory distress, motor impairment, and structural abnormalities in muscles caused by the loss of MEGF10 function. In this study, we deployed cellular and molecular assays to obtain additional insights about *MEGF10*-related myopathy in juvenile, young adult, and middle-aged *Megf10* knockout (KO) mice. We found fewer muscle fibers in juvenile and adult *Megf10* KO mice, supporting published studies that MEGF10 regulates myogenesis by affecting satellite cell differentiation. Interestingly, muscle fibers do not exhibit morphological hallmarks of atrophy in either young adult or middle-aged *Megf10* KO mice. We next examined the neuromuscular junction (NMJ), in which MEGF10 has been shown to concentrate postnatally, using light and electron microscopy. We found early and progressive degenerative features at the NMJs of *Megf10* KO mice that include increased postsynaptic fragmentation and presynaptic regions not apposed by postsynaptic nicotinic acetylcholine receptors. We also found perisynaptic Schwann cells intruding into the NMJ synaptic cleft. These findings strongly suggest that the NMJ is a site of postnatal pathology in *MEGF10*-related myopathy. In support of these cellular observations, RNA-seq analysis revealed genes and pathways associated with myogenesis, skeletal muscle health, and NMJ stability dysregulated in *Megf10* KO mice compared to wild-type mice. Altogether, these data provide new and valuable cellular and molecular insights into *MEGF10*-related myopathy.

**Supplementary Information:**

The online version contains supplementary material available at 10.1186/s13395-024-00342-6.

## Background

*MEGF10*-related myopathy is a rare disorder of limb weakness, difficulty with ambulation, and respiratory dysfunction caused by loss-of-function mutations in *MEGF10* (multiple EGF-like domains 10) [1]. This myopathy generally involves decreased and more variable muscle fiber size as well as other structural disruptions to muscles such as minicores and fibrosis [2, 3]. While it is known that *Megf10* is expressed by muscle satellite cells [4] and glial cells of the neuromuscular junction (NMJ) [1, 5], the cellular and molecular origin and timeline of muscle pathology due to loss of *Megf10* has not been thoroughly investigated.

MEGF10 is a transmembrane protein, the intracellular domain of which signals with NOTCH1 in activated satellite cells to promote their self-renewal [4, 6]. Loss of *Megf10* leads to precocious differentiation of satellite cells [4], suggesting that satellite cells are depleted in *Megf10* deficient muscles [7]. In support of this idea, one study found that a human patient with *MEGF10*-related myopathy had reduced satellite cell numbers [1] and other studies have found that mice with deletion of *Megf10* have impaired muscle regeneration following barium chloride muscle injury [6, 8]. However, studies have not characterized how precocious satellite cell differentiation due to loss of *Megf10* negatively impacts muscle health over time. Furthermore, there is evidence that additional factors are at play in the pathogenesis of *MEGF10*-related myopathy beyond impaired muscle regenerative potential; motor function is impaired in young adult *Megf10* knockout mice despite histology not showing signs of extensive muscle atrophy at this age [6].

An unexplored potential site of pathology in *MEGF10*-related myopathy is the NMJ, where MEGF10 protein has been found to localize in adult mice [1]. While fully differentiated muscle expresses *Megf10* at low levels [4], studies have shown that *Megf10* is expressed by the synaptic glia at the NMJ, known as perisynaptic Schwann cells (PSCs) [5, 9]. MEGF10 is important for the innervation of retinal interneurons [10], suggesting a potential role for MEGF10 in the proper innervation of muscles. MEGF10 is also used by glia such as astrocytes [11, 12] and satellite glial cells [13] for phagocytosis and migration, suggesting that MEGF10 in PSCs might be important for the maintenance or repair of the NMJ. As the proper functioning of the NMJ is vital for preserving muscle health and function [14], if the NMJ is perturbed in *MEGF10*-related myopathy it may contribute to the muscle pathology of this disease.

In this study, we investigated cellular and molecular changes to muscles and NMJs caused by loss of *Megf10*. We examined the structure of muscles and NMJs in male and female *Megf10* constitutive knockout (KO) mice at the juvenile, young adult, and middle-aged time points. We found that muscle mass was decreased in *Megf10* KO mice due to a decrease in fiber number and fiber size growth. However, we found no evidence that skeletal muscles atrophy at these ages. Additional analysis showed that the NMJ is a site of pathology in *Megf10* KO mice. We identified morphological abnormalities in the postsynaptic and presynaptic regions of the NMJ as well as in PSCs that have been found in aged and disease-afflicted NMJs. These findings suggest that MEGF10 may have important functions locally at the NMJ in addition to its role in satellite cells. In support of these cellular observations, transcriptomic analysis uncovered altered expression of genes related to muscle health, myogenesis, and synaptic stress in *Megf10* KO mice. Altogether, this study identifies novel cellular and molecular alterations in muscles and at NMJs of *Megf10* knockout mice that could potentially be used to develop treatments for *MEGF10*-related myopathy.

## Methods

### Animals

*Megf10*tm1b(KOMP)Jr mice [15] with homozygous knockout of the fourth exon are referred to as *Megf10* KO mice. S100β-GFP (B6;D2-Tg(S100b-EGFP)1Wjt/J) [16] and NG2-DsRed mice (Tg(Cspg4-DsRed.T1)1Akik/J) [17] were purchased from Jackson Labs (Bar Harbor, ME) and crossed to generate S100β-GFP; NG2-DsRed mice. Juvenile mice were 1 month old, young adult mice were 3–7 months old, and middle-aged mice were 10–14 months old, with the particular ages of mice used for each experiment described in the figure legends. For some experiments, *Megf10* KO mice were raised and sacrificed at Duke University and then shipped to Brown University for analysis. Other *Megf10* KO mice were bred, raised, and analyzed at Brown University. S100β-GFP; NG2-DsRed mice were raised and sacrificed at Brown University. All of the mice housed at Duke University or Brown University were housed in a 12 h light-dark cycle with *ad libitum* access to water and food. All experiments were carried out under NIH guidelines and those of the Institutional Animal Care and Use Committees of Brown University (Protocol# 22-09-0003) and Duke University (Protocol# A211-21-10).

### Immunohistochemistry

Antibodies used for immunohistochemistry (IHC) include: rabbit anti-synaptophysin antibody (1:100, Invitrogen, 180,130), rabbit anti-S100β antibody (1:400, Dako, Z0311), mouse IgG1 anti-neurofilament antibody (1:400, DSHB, 2H3-s), mouse IgG1 anti-SV2 antibody (1:400, DSHB, SV2- s), mouse IgG1 anti-NeuN antibody (1:500, Millipore Sigma, MAB377), guinea pig anti-VAChT antibody (1:1000, Millipore Sigma, AB1588), Alexa Fluor 488 conjugated polyclonal goat anti-mouse IgG1 (1:500, Thermo Fisher, A21121), Alexa Fluor 488 conjugated polyclonal goat anti-guinea pig (1:500, Thermo Fisher, A11073), Alexa Fluor 555 conjugated polyclonal goat anti-mouse IgG1 (1:500, Thermo Fisher, A21127), and Alexa Fluor 568 conjugated polyclonal goat anti-rabbit IgG (1:500, Invitrogen, 2,599,544). Alexa Fluor 488 conjugated alpha-bungarotoxin (1:500, Thermo Fisher, B13422) and Alexa Fluor 647 conjugated alpha-bungarotoxin (1:500, Invitrogen, B35450) were used to label nAChRs. Rhodamine-WGA (1:500, Vector Laboratories, RL-1022) was used to outline muscle fibers in cross section. DAPI (1:1000, Thermo Fisher, D1306) was used to label nuclei.

Muscles were dissected from mice following transcardial perfusion with 4% paraformaldehyde. For IHC of muscle cross-sections, tibialis anterior (TA) muscles were incubated in 30% sucrose in PBS overnight at 4 °C, cut in half perpendicular to their length with a razor, embedded in Tissue-Tek OCT compound (Sakura), and cross-sectioned at 16 μm with a cryostat. Muscle cross sections were placed on gelatin-coated glass microscope slides, washed in PBS, incubated in blocking buffer (5% BSA, 3% goat serum in PBS) for 1 h at room temperature and then incubated in WGA-Rhodamine and DAPI in blocking buffer for 1 h at room temperature, washed 3x in PBS, and covered in Vectashield (Vector Laboratories, H- 1000) before coverslip application. For IHC of NMJs, the extensor digitorum longus (EDL), soleus, diaphragm, and triangularis muscles were dissected and some were post-fixed for 1 min in ice-cold methanol and then washed 3x in PBS. Then, all of the muscles were incubated in blocking buffer (5% BSA, 3% goat serum, 0.5% Triton X-100 in PBS) for 1 h at room temperature, incubated in primary antibody diluted in blocking buffer overnight at 4 °C, washed 3x in blocking buffer, incubated in secondary antibody, DAPI and fBTX for 3 h at 4 °C, washed 3x in PBS, and mounted to microscope slides in Vectacshield. For IHC of motor neurons, spinal cords were dissected and immediately post-fixed in 4% paraformaldehyde for 2 h at 4 °C, washed 3x in PBS, incubated in 30% sucrose in PBS overnight at 4 °C, embedded in Tissue-Tek OCT compound, and the lumbar region was cross-sectioned at 50 μm with a cryostat. Spinal cord cross sections were placed on gelatin-coated glass microscope slides, washed in PBS, incubated in blocking buffer (5% BSA, 3% goat serum, 0.1% Triton X-100 in PBS) for 1 h at room temperature, incubated in primary antibody diluted in blocking buffer overnight at 4 °C, washed 3x in blocking buffer, incubated in secondary antibody for 3 h at 4 °C, washed 3x in PBS, and covered in Vectashield before coverslip application.

### Confocal Microscopy

Confocal images were obtained with a Zeiss LSM 900 laser scanning confocal microscope (Carl Zeiss Microscopy, Berlin, Germany) equipped with 405, 488, 561, and 640 nm lasers using a 20 × (0.8 numerical aperture) or a 63 × (1.4 numerical aperture) objective. Maximum intensity projections and stitching of tile scans were generated using Zeiss Zen Black software.

### Confocal image analysis

All confocal image analysis was performed in ImageJ software (version 2.1.0/1.53c). For all analyses, the genotype (and sex, when appropriate) was blinded to the analyst. Muscle fiber analysis was performed on TA cross-sections stained with fWGA/DAPI, with 4 cross sections analyzed per mouse. Muscle fiber number was counted by hand from a single TA cross section. Muscle fiber size and central nuclei prevalence were measured using an average of 355 fibers randomly sampled across the 4 cross sections using the fractionator method [18]. Muscle fiber size was measured by outlining each muscle fiber using the polygon tool in ImageJ to measure minimum Feret’s diameter. These fibers were identified as having a central nucleus if the muscle fiber had at least one DAPI-labeled nucleus within the muscle fiber (i.e. not touching the edge of the fWGA stained muscle fiber).

NMJ presynapse and postsynapse analyses were performed on whole mounted EDL, soleus, and diaphragm muscles stained for synaptophysin and with fBTX labeling of nAChRs. Postsynaptic fragmentation was measured as the number of discrete, non-touching fBTX + nAChR islands per NMJ, with an average of 49 NMJs analyzed per mouse. The following analyses were performed on the diaphragm only, with an average of 12 NMJs analyzed per mouse. Receptor area was determined by measuring the area of fBTX + pixels at a given NMJ following signal thresholding in ImageJ. Dispersion index was captured at the same time in ImageJ by collecting Shape Descriptors which includes Solidity, which is the area of fBTX staining divided by the area of a perimeter around all of the fBTX staining. Junctional area, which is the area of the perimeter around the fBTX + AChR islands, was then derived by dividing the receptor area by the dispersion index. Innervation was determined by measuring the total area of fBTX signal for each NMJ and subtracting the area of fBTX AChRs lacking an apposing synaptophysin + axon terminal (i.e. subtracting the area of denervated AChRs), and then dividing these values. Postsynaptic coverage was determined by measuring the total area of the synaptophysin + axon terminal and subtracting the area of synaptophysin + axon terminal lacking opposing fBTX + AChRs, and then dividing these values.

PSC number and morphology analyses were performed on whole mounted triangularis muscles stained for S100β, SV2, and neurofilament, and with fBTX/DAPI, with an average of 23 NMJs analyzed per mouse. NMJs were selected for imaging that were en face and near the surface of the muscle tissue, as this allows best imaging of NMJ morphology. PSCs were counted per NMJ using the counter tool in ImageJ, being identified as S100β-positive cells with a nucleus/cell body apposed to postsynaptic nAChRs. PSC sprouts were identified as extensions of PSCs away from the NMJ that are at least 3 μm long using the line selection tool in ImageJ, as described previously [5]. Migrating Schwann cells (SCs) were identified as S100β-positive cells that have a cell body which is not apposed to the postsynaptic nAChRs but have sprouts which are both continuous with the rest of the PSC processes over the postsynapse and extending away from the NMJ, as described previously [5].

Motor neuron number and size analyses were performed on lumbar spinal cord cross sections stained for NeuN and VAChT. Measurements were collected from both ventral horns of a single spinal cord cross section per mouse and then averages were taken between them. Alpha motor neurons were counted per ventral horn using the counter tool in ImageJ, identified as large NeuN + cells in the ventral horn of the spinal cord with VAChT + punctae along the soma and dendrites. The large size of alpha motor neurons and their location within the ventral horn was used to distinguish alpha motor neurons from other cholinergic neurons. Soma size was measured per alpha motor neuron using the polygon tool in ImageJ in the Z-slice in which the soma size was greatest, with an average of 20 motor neurons analyzed per mouse. Edge artifacts were avoided for this analysis by only analyzing alpha motor neuron that were fully captured in the Z-stack.

### Transmission Electron Microscopy

One 6-month-old female wild-type mouse and one 6-month-old female *Megf10 KO* mouse were perfused transcardially with 0.1 M sodium cacodylate buffer with 2mM calcium chloride and 100mM sucrose at pH 7.4 at room temperature, followed by the same buffer with 2% PFA and 3% glutaraldehyde. The smallest segment of the EDL was immediately dissected and then fixed overnight at room temperature in 2% PFA, 3% glutaraldehyde in buffer. The EDL segments were cut in half perpendicular to their length with a razor blade near the endplate band. The muscle pieces were washed in buffer and then stained in 1% osmium tetroxide (Sigma Aldrich, 75,632), 1% ferrocyanide (Sigma Aldrich, P3289) in buffer for 5 h at room temperature. The muscles were then washed in ddH2O and then stained with 1% uranyl acetate for 2 h at room temperature. Muscles were washed in ddH2O and then dehydrated in graded ethanol at 30%, 50%, 70%, 90%, 95%, and 100% ethanol for 20 min at each step. After three 10-minute washes in 100% ethanol, the muscles were embedded in SPURR Low Viscosity Embedding Kit, Hard Mix (EMS, Cat #14,300). A Leica EM UC7 Ultramicrotome was used to trim the blocks with a razor blade and then a diamond knife, and then a diamond knife was used to obtain approximately 20 100 nm cross sections per sample. Then, to sample at different depths within the tissue and image different NMJs, 45 μm of tissue was discarded and then another 20 100 nm cross sections were collected per sample. These sections were mounted on bare 200 mesh or 300 mesh copper grids, which were then imaged by a Philips EM410 Transmission Electron Microscope with a NANOSPRT5 camera and AMT V701 software. Digital images of muscle fibers and NMJs were captured from sections at magnifications between 21,000x and 38,000x. In total, images of 28 young adult wild-type NMJs and 29 young adult Megf10 KO NMJs were taken. It was not possible to determine which of these images were of different NMJs or whether images were of the same NMJ at different depths, but imaging at two different depths within the tissue increases the likelihood that we sampled several different NMJs per mouse. For analysis of axon terminal synaptic vesicles and mitochondria, 37 wild-type and 21 Megf10 KO synaptic regions with the best delineation of these ultrastructural features were analyzed from these NMJs. A synaptic region is defined as a synaptic gutter and its apposing axon terminal. The muscle, axon terminal, and PSCs in each image were identified by morphology. Adobe Photoshop 2022 (version 23.2) was used to pseudocolor each of these cell types red, blue, and green, respectively.

Transmission electron micrographs were analyzed in ImageJ. PSC processes intruding in the synaptic cleft were identified as NMJs with PSC processes that extended at least 200 nm into the synaptic cleft. Loss of junctional folds was identified as NMJs with at least 20% of the postsynapse lacking junctional folds. Synaptic vesicle density and mitochondria density were calculated for motor axon terminals in which these structures were easily identifiable by dividing counts of synaptic vesicles and mitochondria by the area of the motor axon terminal which was measured using the polygon tool in ImageJ. Mitochondria density was calculated for muscle postsynaptic regions in which these structures were easily identifiable by dividing counts of mitochondria by the area of an arch-shaped region emanating 1 μm from the synaptic cleft into the muscle which was measured using the polygon tool in ImageJ.

### Fluorescence activated cell sorting (FACS) of PSCs

Young adult (4–5 month old) female S100β-GFP; NG2-DsRed mice were sacrificed and the hindlimb muscles were immediately collected and dissociated in 2 mg/mL collagenase II (Worthington Chemicals, Lakewood, NJ) followed by mechanical trituration. A single cell suspension was created with a 40 μm filter, and then excess debris was removed by centrifugation in 4% BSA followed by centrifugation in 40% Optiprep solution (Sigma-Aldrich, St. Louis, MO) from which the interphase was collected. A BD FACS Melody Cell Sorter (BD Biosciences) was used to perform FACS. Each replicate was a single mouse, and approximately 5,000 PSCs and 30,000 other SCs were collected per replicate.

### qPCR

RNA was isolated from whole soleus muscle or from FACS-isolated PSCs and other SCs. RNA was isolated from whole muscle by Azenta. RNA was isolated from the PSCs and other SCs using the PicoPure RNA Isolation Kit (Thermo Fisher). All of the RNA was then reverse transcribed with iScript (BioRad, Hercules, CA). The cDNA from isolated PSCs and other SCs was preamplified with SsoAdvanced PreAmp Supermix (Bio-Rad, 1,725,160). Then, qPCR was performed on all samples with iTAQ SYBR Green Supermix (Bio-Rad, 1,725,121) using 300 nM primers for *Megf10* (F: CAACTCCAGCCAACAGGAATG, R: GCAGCAGGTCATAATGGCAAG), *Fgfbp1* (F: ACACTCACAGAAAGGTGTCCA, R: CTGAGAACGCCTGAGTAGCC), *Pax7* (F: GCGAGAAGAAAGCCAAACAC, R: GTCGGGTTCTGATTCCACAT), *Chrng* (F: GCTCAGCTGCAAGTTGATCTC, R: CCTCCTGCTCCATCTCTGTC), *Myh3* (F: CTTCACCTCTAGCCGGATGGT, R: AATTGTCAGGAGCCACGAAAAT), and *Gapdh* (F: CCCACTCTTCCACCTTCGATG, R: GTCCACCACCCTGTTGCTGTAG) on a CFX Connect Real Time PCR System (Bio-Rad). Expression values were normalized to *Gapdh* using the 2-ΔΔCT method.

### RNA-seq and analysis

Female 7-month-old WT and *Megf10* KO mice were euthanized and the soleus muscle was immediately dissected and frozen in liquid nitrogen. RNA isolation and bulk RNA-seq were subsequently performed by Azenta with 4 replicates per genotype (i.e. WT and *Megf10* KO) at a sequencing depth of 30 million reads per sample. Trimming of the RNA-seq data for quality and to remove adaptors was performed using Trimmomatic v.0.36 [19] and the Nextera TruSeq paired-end adapter (TruSeq3-PE-2.fa). Then, transcripts were indexed, aligned, and quantified by Salmon v.0.11.3 [20]. Examination of QC summary statistics was performed using FastQC v0.11.5 and MultiQC v1.0 [21]. After alignment, R statistical software v4.1.2 was used to generate lists of differentially expressed genes. Ensemble transcript IDs were converted to gene IDs by Biomart [22]. Salmon quantification files were imported using Tximport [23]. Differentially expressed genes were determined by DESeq2 [24]. Count reads of 5 or less were filtered out before running DESeq2. EnhancedVolcano was used to generate volcano plots [25]. Functional and pathway analysis was performed using Ingenuity Pathway Analysis (QIAGEN Inc, https://www.qiagenbio-informatics.com/products/ingenuity-pathway-analysis). The computational resources and services at the Center for Computation and Visualization, Brown University, supported this analysis.

### Statistics

Comparisons between two groups were made using an unpaired Student’s t-test or Welch’s unpaired Student’s t-test, depending on the results of an F-test of variance. When the groups in the experiment had more than two variables, comparisons were made with a two-way ANOVA and Tukey’s post-hoc analysis. GraphPad Prism (Version 9.5.1) was used for all statistical analyses and graph generation. A biological replicate was defined as a single mouse for all experiments except electron microscopy analysis where a biological replicate was defined as a single NMJ cross section. Biological replicate numbers are provided in the figure legends. Data are expressed as mean + standard deviation.

## Results

### Fewer muscle fibers are formed in *Megf10* KO mice

**Fig. 1:**
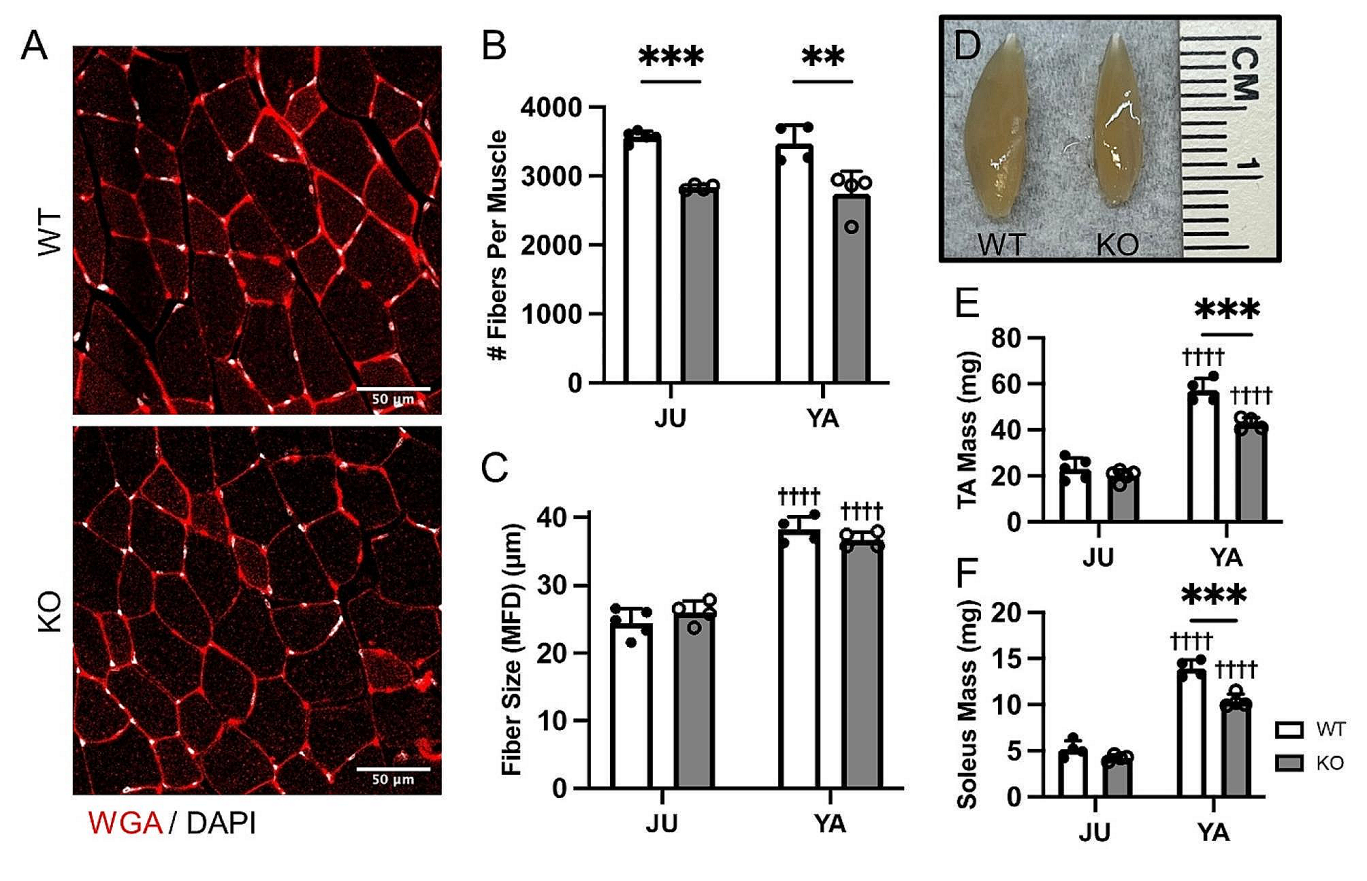
Muscle fiber formation is impaired in *Megf10* KO mice. (**A**) Representative images of tibialis anterior (TA) muscle cross sections from young adult male WT and *Megf10* KO mice. TA cross sections from juvenile and young adult male WT and *Megf10* KO mice were examined for (**B**) fiber number per TA and (**C**) fiber size (minimum Feret diameter (MFD)). (**D**) Representative image of isolated TA muscles from young adult male WT and *Megf10* KO mice. Whole muscle mass was recorded for the (**E**) TA and (**F**) soleus muscles. Two-way ANOVA with Tukey’s multiple comparison test for statistical analysis. Values represented as mean + SD. **p < 0.01, ***p < 0.001 versus WT. ^††††^*p* < 0.0001 versus juvenile. JU (juvenile, 1 mo), YA (young adult, 3 mo)

Patients with *MEGF10*-related myopathy have decreased muscle mass and strength [1, 26] accompanied by decreased muscle fiber size and structural abnormalities [1, 2]. Studies of *Megf10* knockout (KO) mice have demonstrated that these pathologies may at least sprout due to the depletion of satellite cells [6, 8]. Yet it is not known how loss of *Megf10* impacts the formation, growth, and eventual atrophy of muscle fibers. To better understand this process, we first examined muscles from juvenile (1-month-old) and young adult (3-month-old) male WT and *Megf10* KO mice. We observed a ∼ 20% decrease in muscle fiber number in the tibialis anterior (TA) muscle of juvenile *Megf10* KO mice which persisted into young adulthood (Fig. 1A-B). However, this occurred in the absence of muscle atrophy. Muscle fibers were similar in size (Fig. 1C), size distribution (Fig. S1A), and location of their myonuclei (Fig. S1C), in the TA muscles of *Megf10* KO mice compared to age- and sex-matched control mice. Consistent with the TA in young adult *Megf10* KO mice having fewer muscle fibers, the TA mass is smaller in *Megf10* KO mice compared to control mice (Fig. 1D-E). The soleus mass is also reduced (Fig. 1F), but not the mass of the extensor digitorum longus (EDL) (Fig. S2A) in young adult *Megf10* KO mice.

### Muscle fiber growth is attenuated in adult *Megf10* KO mice

**Fig. 2:**
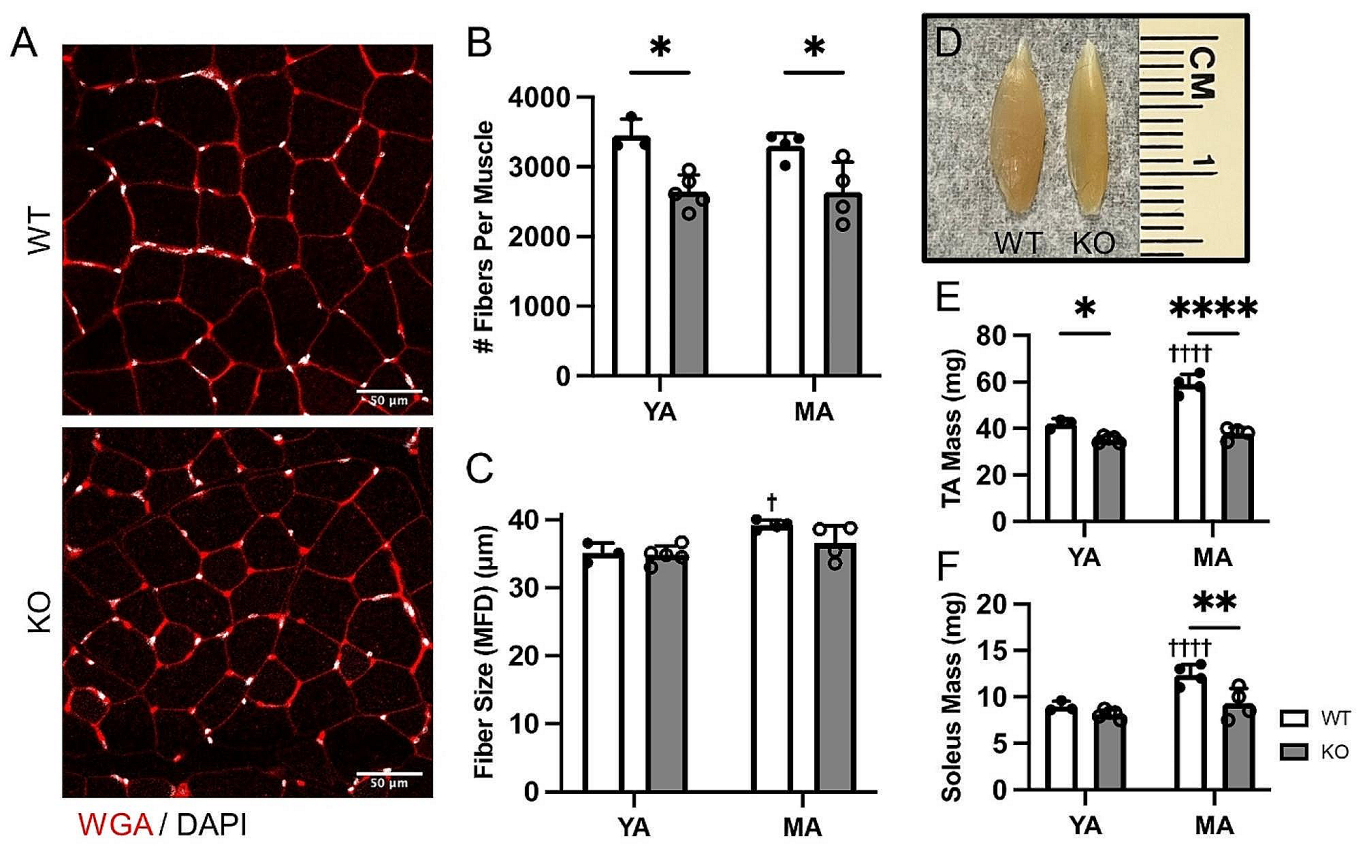
Muscle fiber growth is decreased in *Megf10* KO mice. (**A**) Representative images of tibialis anterior (TA) muscle cross sections from middle-aged female WT and *Megf10* KO mice. TA cross sections from young adult and middle-aged female WT and *Megf10* KO mice were examined for (**B**) fiber number per TA and (**C**) fiber size (minimum Feret diameter (MFD)). (**D**) Representative image of isolated TA muscles from middle-aged female WT and *Megf10* KO mice. Whole muscle mass was recorded for the (**E**) TA and (**F**) soleus muscles. Two-way ANOVA with Tukey’s multiple comparison test for statistical analysis. Values represented as mean + SD. *p < 0.05, **p < 0.01, ****p < 0.0001 versus WT. ^†^*p* < 0.05, ^††††^*p* < 0.0001 versus young adult. YA (young adult, 3 mo), MA (middle-aged, 12–14 mo)

Next, we assessed whether *Megf10* KO muscles at later ages accrued additional pathological features such as muscle atrophy or impaired muscle growth. For this, we examined muscles from young adult (3-month-old) and middle-aged (12-14-month-old) female WT and *Megf10* KO muscles. Similar to the younger time points, we found a consistent ∼ 20% decrease in muscle fiber number (Fig. 2A-B) and no change in indicators of muscle atrophy in middle-aged *Megf10* KO mice (Fig. 2C) (Fig S1B, D). Electron micrographs showed no additional pathological features in myofibrils, sarcoplasmic reticulum, myonuclei, or muscle mitochondria in *Megf10* KO muscle (Fig. S3). It is important to note that fiber size increased between young adulthood and middle age in WT mice but not in *Megf10* KO mice (Fig. 2C), suggesting that muscle fiber growth is impaired in *Megf10* KO mice transitioning from young adulthood to middle-age.

Consistent with a decrease in fiber number and fiber growth, the TA mass is reduced in young-adult and middle-aged *Megf10* KO mice compared to WT mice (Fig. 2D-E). Interestingly, the TA mass did not increase in mass as *Megf10* KO mice transition into middle-age, in stark contrast to the TA in WT mice (Fig. 2D-E). Similar patterns were observed in the soleus (Fig. 2F) but not in the EDL (Fig. S2B) muscles. The lower muscle mass may explain why middle-aged *Megf10* KO mice weight less than WT mice (Fig. S4). Together, these results suggest that loss of *Megf10* impairs muscle fiber formation during development and muscle fiber growth in adulthood but does not cause significant muscle atrophy. The similarities in EDL mass between WT and *Megf10* KO mice suggest that the impact of *Megf10* deletion on muscle fiber formation and fiber growth may not be consistent across all muscles.

### NMJs prematurely acquire age- and disease-related features in *Megf10* KO mice

**Fig. 3:**
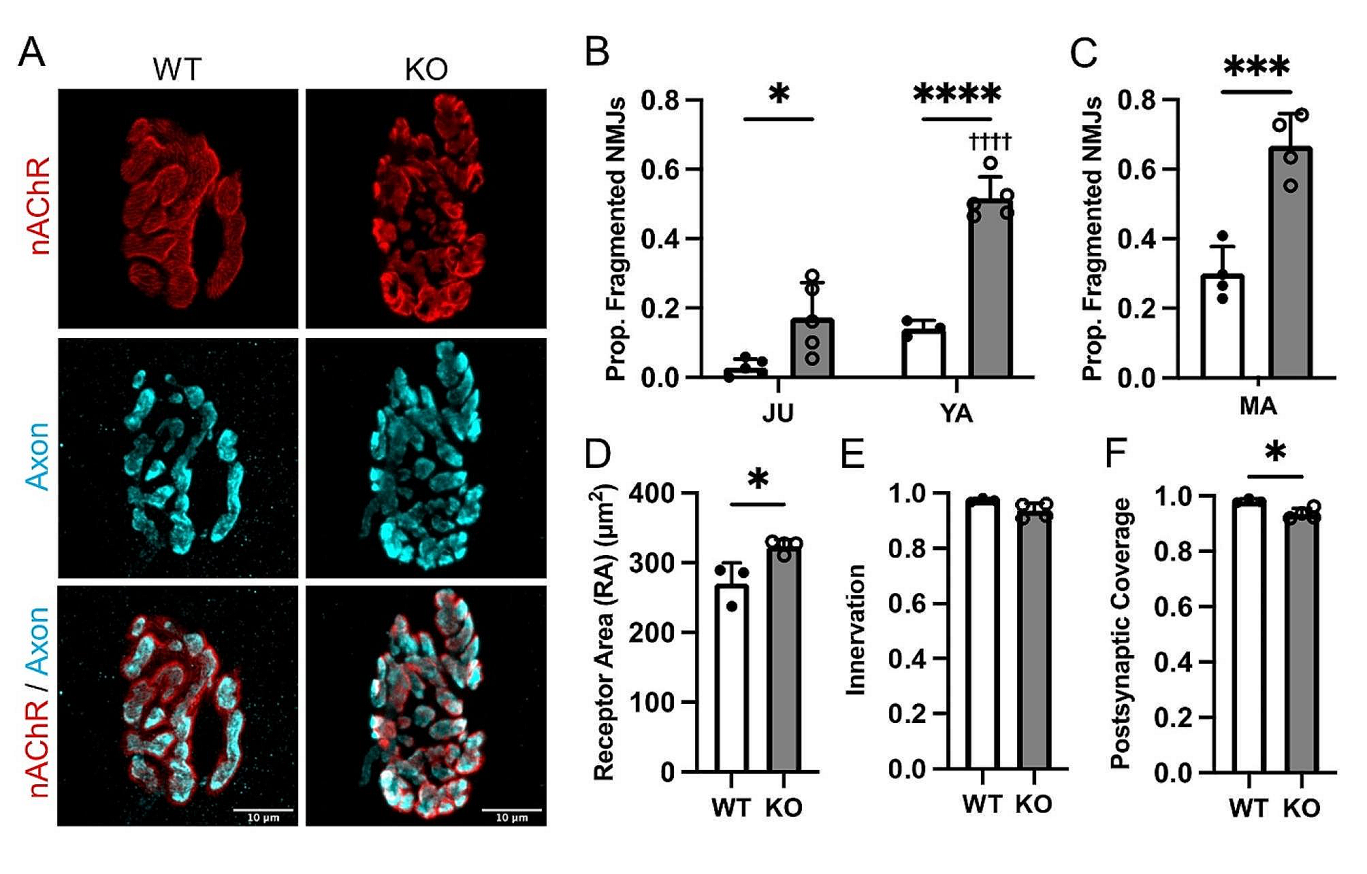
NMJ postsynaptic fragmentation and impaired apposition of the pre- and postsynapse in *Megf10* KO mice. (**A**) Representative images of the NMJ presynapse labeled against synaptophysin (cyan) and and postsynaptic nicotinic acetylcholine receptors (nAChRs) labeled with fBTX (red) from male juvenile WT and *Megf10* KO diaphragm muscles. (**B**) Male and (**C**) female postsynapses were analyzed for the proportion NMJs with postsynaptic fragmentation (> 4 distinct nAChR islands). Young adult male diaphragms were then analyzed for postsynaptic (**D**) receptor area (area of nAChRs), (**E**) junctional area (area of a perimeter around the nAChRs), and (**F**) dispersion index (receptor area / junctional area). In young adult male diaphragms, the apposition of the pre- and postsynaptic stains for the NMJ was assessed to determine the (**G**) innervation status (proportion of the postsynaptic nAChRs innervated by the motor axon terminal) and (H) postsynaptic coverage (proportion of the motor axon terminal apposed by postsynaptic nAChRs). Two-way ANOVA with Tukey’s multiple comparison test (B) or unpaired two-sided Student’s t-test (C-H) for statistical analysis. Values represented as mean + SD. *p < 0.05, **p < 0.01, ***p < 0.001, ****p < 0.0001 versus WT. ^††††^*p* < 0.0001 versus juvenile. JU (juvenile, 1mo), YA (young adult, 3 mo), MA (middle-aged, 12-14 mo)

While there is evidence that MEGF10 is enriched at the NMJ in adult mice [1], it has yet to be determined if NMJ degeneration occurs in *Megf10* KO mice or in patients with *MEGF10*-related myopathy. To shed light on this, we examined the impact of *Megf10* deletion on NMJ integrity in mice. We observed robust structural changes in NMJ postsynapses that are associated with muscular dystrophies, aging, and amyotrophic lateral sclerosis (ALS) [14, 27] in the diaphragm muscle of juvenile, young adult, and middle-aged *Megf10* KO mice. These included a progressive increase in postsynaptic fragments (Fig. 3A-C), area occupied by nAChRs (Fig. 3D), total endplate area (Fig. S5A), and dispersion of nAChRs (Fig. S5B) in *Megf10* KO mice. Increases in postsynaptic fragments were also observed in the soleus of *Megf10* KO mice in the young adult and middle-aged groups but only in the young adult age group for the EDL (Fig. S6). While *Megf10* KO NMJs were innervated to a similar extent as WT NMJs in the diaphragm (Fig. 3E), there was a decrease in the apposition between the pre- and postsynapse such that there were more presynaptic regions not apposed by the postsynapse (Fig. 3F). That NMJs acquire structural abnormalities in otherwise healthy muscle fibers suggest that MEGF10 plays important roles specifically at the NMJ.

### Electron microscopy reveals additional cellular abnormalities in NMJs of *Megf10* KO mice

**Fig. 4:**
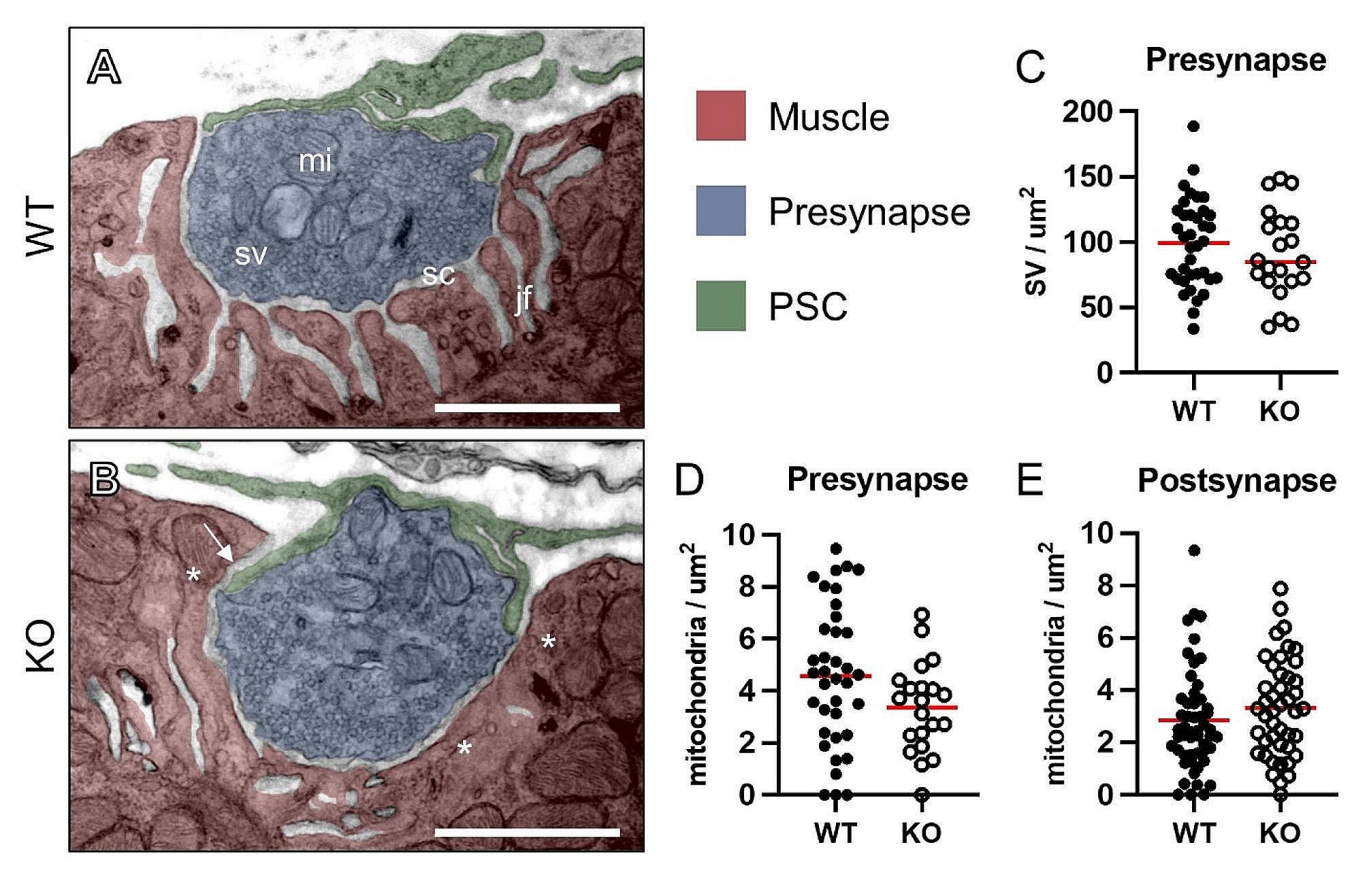
Ultrastructural abnormalities in *Megf10* KO NMJs. (**A-B**) Representative transmission electron micrographs of NMJs in cross sections from young adult (6 mo) female WT and *Megf10* KO extensor digitorum longus (EDL) muscles. (A) WT NMJs with the labels for synaptic vesicles (sv), the synaptic cleft (sc), mitochondria (mi), and junctional folds (jf). (B) *Megf10* KO NMJ showing loss of junctional folds on the postsynapse (*) and intrusion of PSC processes into the synaptic cleft (arrow). Density of (**C**) synaptic vesicles in the presynapse, (**D**) mitochondria in the presynapse, and (**E**) mitochondria in the postsynapse are quantified, with red horizontal lines indicating means. Scale bars are 1 μm. *n* = 1, ∼ 30 NMJs analyzed per mouse

Transmission electron micrographs (TEMs) from young adult (6-month-old) WT and *Megf10* KO EDL cross sections uncovered additional NMJ abnormalities in the postsynapse, presynapse, and PSC components of the NMJ (Fig. 4A-B). PSCs intruded more frequently into the synaptic cleft of *Megf10* KO NMJs (WT: 6 of 28, KO: 19 of 29) (Fig. S7). This intrusion is also observed at aging NMJs [5], a cellular alteration suggested to impair neurotransmission at NMJs [28, 29]. TEMs also showed that presynapses of *Megf10* KO mice have decreased synaptic vesicle (SV) density (WT: 99 ± 33.7 SVs/µm^2^, KO: 90.2 ± 33.9 SVs/µm^2^) and mitochondria density (WT: 4.6 ± 2.7 mitochondria/µm^2^, KO: 3.4 ± 1.7 mitochondria/µm^2^) (Fig. 4C-D), which has been noted in a mouse model of spinal muscular atrophy and could indicate a difference in synaptic activity [30]. Additionally, the density of muscle mitochondria situated directly next to the NMJ postsynapse was increased in *Megf10* KO mice (WT: 2.8 ± 2.0 mitochondria/µm^2^, KO: 3.4 ± 1.9 mitochondria/µm^2^) (Fig. 4E), which is a change that has not been previously described, to our knowledge, but may indicate increased metabolic demand of the NMJ on the muscle. We also observed more postsynaptic regions lacking junctional folds in *Megf10* KO mice (WT: 11 of 28, KO: 18 of 29), an NMJ phenotype seen in aged mice [5] and laminin-β2 deficient mice [29].

### PSC numbers are unchanged in *Megf10* KO mice

**Fig. 5:**
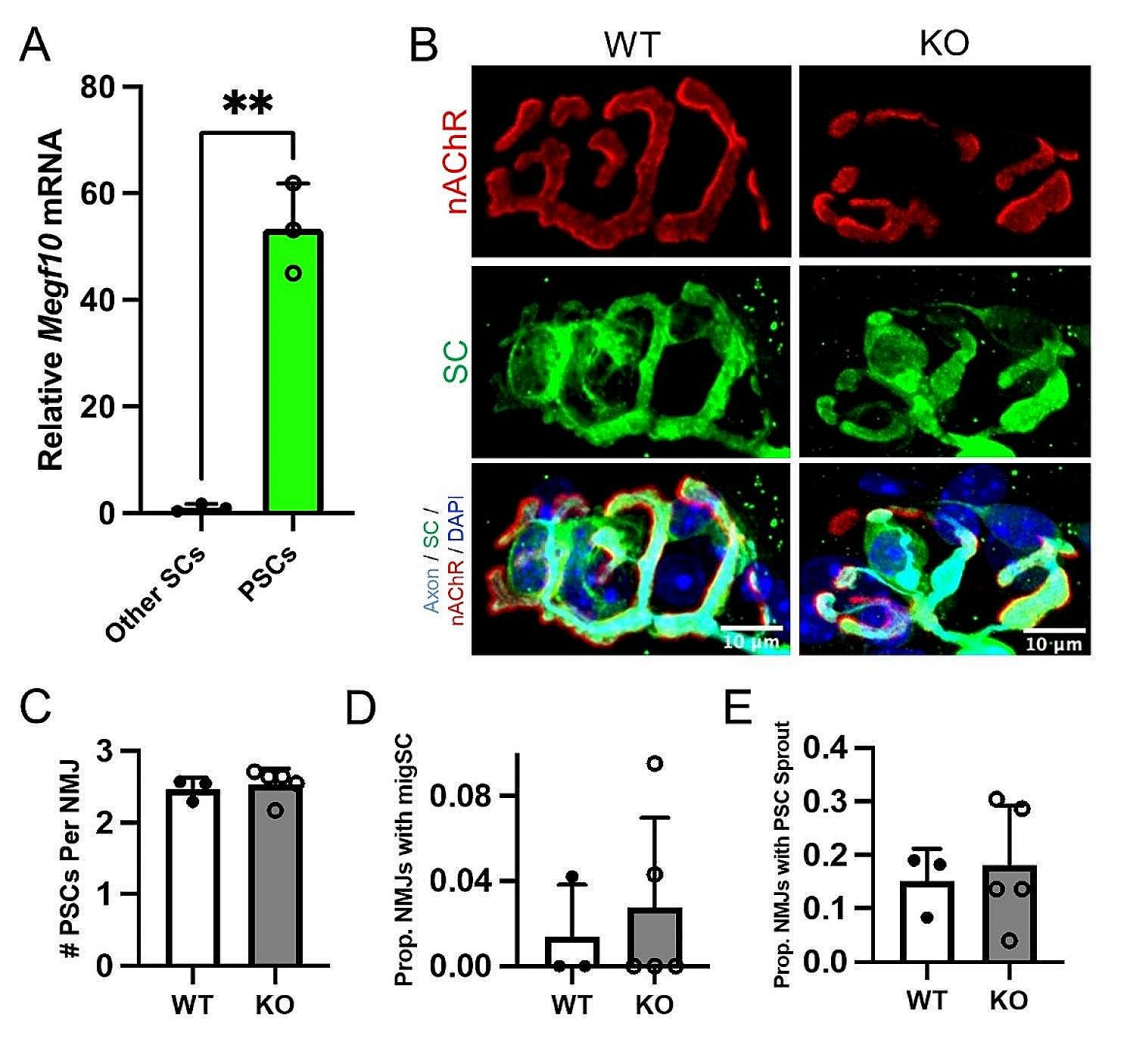
*Megf10* KO NMJs do not have disrupted PSC number or sprouting. (**A**) qPCR for *Megf10* on FACS-isolated PSCs and other SCs from young adult (4–5 mo) female S100B-GFP; NG2-dsRed mice. (**B**) Representative images of NMJ postsynapses stained with fBTX (red), Schwann cells stained against S100B (green), presynapses and axons stained against SV2 and neurofilament (cyan), and postsynapses stained with DAPI (blue) from young adult (3 mo) male WT and *Megf10* KO triangularis. PSCs are identified as S100B + Schwann cells overlying the NMJ postsynapse. These images were analyzed for (**C**) PSC number per NMJ, (**D**) the proportion of NMJs with a migrating Schwann cell, or (**E**) the proportion of NMJs in which PSCs extend sprouts away from the NMJ. Unpaired 2-sided Student’s t-test for statistical analysis. Values represented as mean + SD. **p < 0.01

MEGF10 has been shown to play important roles in mediating glial functions [11–13]. Our group previously discovered that *Megf10* is enriched in PSCs compared to other Schwann cells (SCs) in skeletal muscles using RNA-seq [5, 31]. We validated these results in PSCs and other SCs isolated from young adult (4-5-month-old) S100-GFP; NG2-DsRed mice using qPCR (Fig. 5A). We next used an antibody against S100B to determine whether *Megf10* deletion leads to age-related characteristics of PSCs such as hyperproliferation, migration away from the NMJ, and formation of sprouts [5]. We found no differences in PSC numbers (Fig. 5B-C), migration (Fig. 5D), or formation of sprouts (Fig. 5E) between young adult (3-month-old) *Megf10*-deficient and WT mice. Further investigation is needed to determine whether *Megf10* plays additional roles in PSCs, such as in mediating their phagocytic activity, tiling, and guidance of innervating motor axons.

### Motor neuron somas are spared in *Megf10* KO mice

**Fig. 6:**
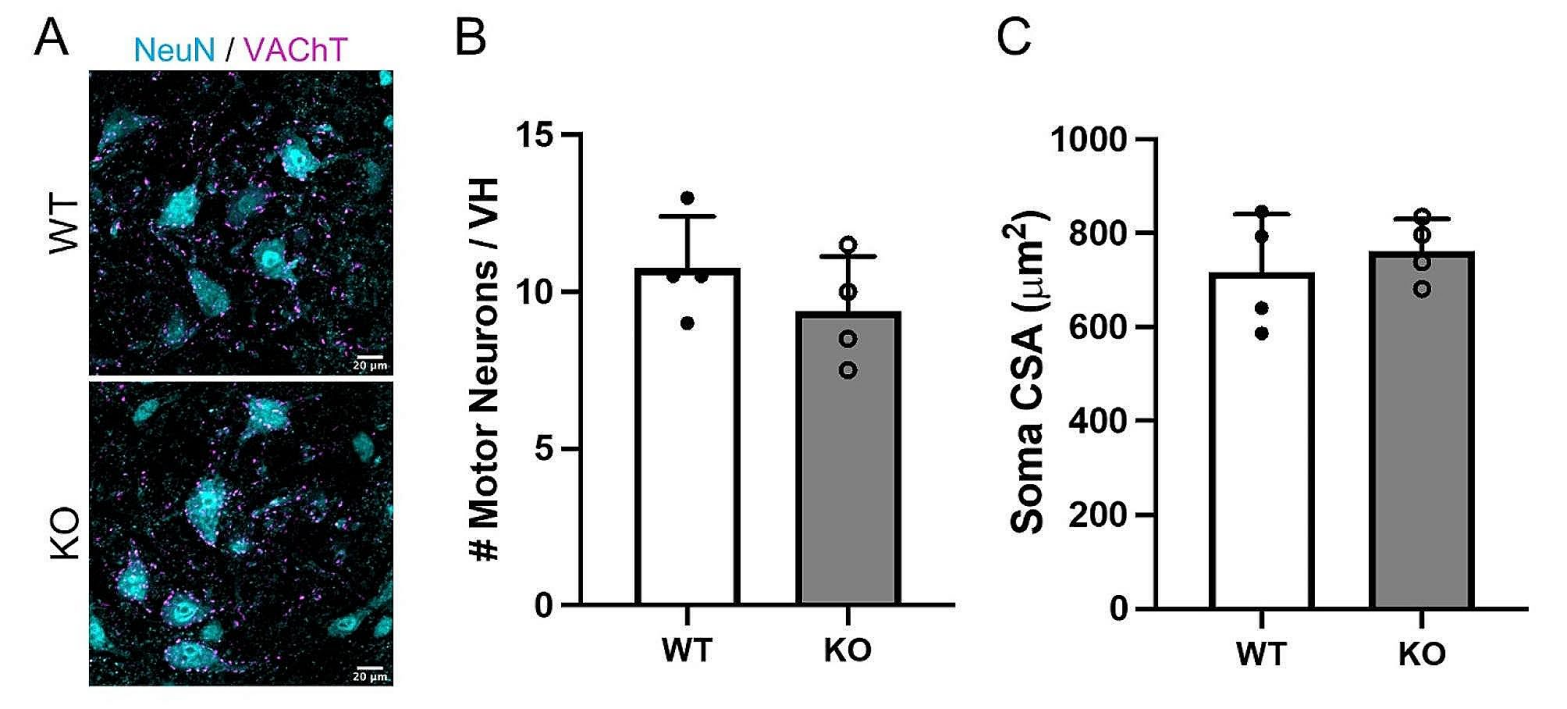
Motor neuron density and soma size are unaffected by *Megf10* KO. (**A**) Representative images of neurons labeled with NeuN (cyan) and cholinergic synapses stained against VAChT (magenta) in cross sections of the ventral horn of the lumbar spinal cord from young adult (3 mo) male WT and *Megf10* KO mice. Alpha motor neurons are identified as large NeuN + cells with cholinergic synapses along their soma and dendrites. Motor neuron (**B**) density and (**C**) soma size (cross sectional area (CSA)) are unaffected in *Megf10* KO mice. Unpaired 2-sided Student’s t-test for statistical analysis. Values represented as mean + SD.

We asked whether the effects of *Megf10* deletion on motor axon terminals, revealed by electron microscopy, extend to the soma. We visualized motor neurons by immunostaining for the pan-neuronal marker, NeuN, and C-boutons, cholinergic inputs that terminate on the soma of motor neurons (Fig. 6A). Our analysis revealed no change in either the number (Fig. 6B) or size (Fig. 6C) of lumbar motor neurons in *Megf10* KO compared to age- and sex-matched WT young adult (3-month-old) mice. Thus, the presynaptic alterations in *Megf10* KO appear to result from either changes locally at the NMJ or elsewhere in skeletal muscles.

### Altered expression of genes associated with myogenesis, muscle stress and the NMJ in *Megf10* KO muscle

**Fig. 7:**
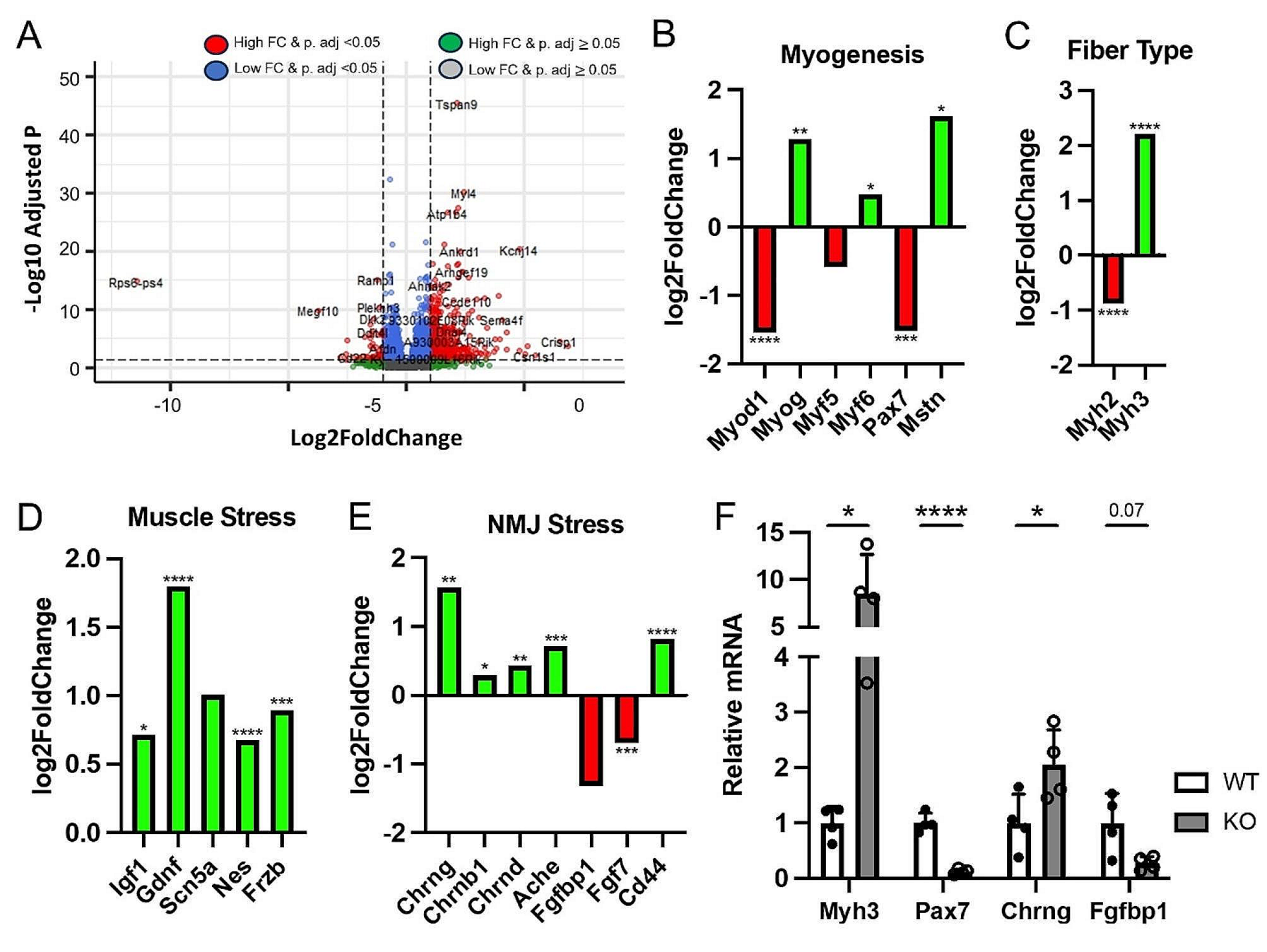
Transcriptional analysis of *Megf10* KO muscle. Bulk RNA-seq was performed on whole soleus muscles isolated from young adult (7 mo) female WT and *Megf10* KO mice (*n* = 4), which (**A**) identified many upregulated and downregulated genes in *Megf10* KO muscle. Differentially expressed genes in *Megf10* KO muscle were related to (**B**) myogenesis, (**C**) muscle fiber type, (**D**) muscle stress, and (E) NMJ stress. Fold changes are displayed as relative expression in *Megf10* KO muscle compared to WT muscle. (**F**) qPCR was used to confirm some of the differentially expressed genes identified by RNA-seq. (A-**E**) DESeq2 or (**G**) unpaired 2-sided Student’s t-test for statistical analysis. Values represented as mean + SD. *p < 0.05, **p < 0.01, ***p < 0.001, ****p < 0.0001

To uncover transcriptional changes that may precipitate the observed changes to muscles and NMJs, we performed bulk RNA-seq on whole soleus muscles from young adult (7-month-old) WT and *Megf10* KO mice. The soleus was chosen due to its robust decrease in muscle mass and increase in NMJ postsynaptic fragmentation in *Megf10* KO mice. Comparison of the transcriptional profile of WT and *Megf10* KO muscle revealed over 2,400 differentially expressed genes, with top altered canonical pathways related to neuroinflammation and *S100* signaling based on Ingenuity Pathway Analysis (Fig. S8).Among these are genes associated with myogenesis, muscle fiber type, and muscle stress. We found *Pax7*, a marker of satellite cells, decreased in *Megf10* KO mice (Fig. 7B) [32]. The expression of other myogenic regulatory factors such as *Myod1*, *Myog*, and *Myf6*/*Mrf4* were also altered in *Megf10* KO muscle (Fig. 7B) [33]. Along with these changes, *Myh2*, a marker of fast twitch type IIa fibers [34] is reduced while *Myh3*, which encodes the embryonic form of myosin and increases upon muscle damage and regeneration [35], is elevated in *Megf10* KO mice (Fig. 7C). We also found altered expression of genes which levels change in denervated muscles, such as *Igf1*, *Gdnf*, *Nes*, and *Frzb* (Fig. 7D) [36–39].

Additionally, our transcriptional analysis revealed NMJ-associated genes altered in *Megf10* KO mice. We found increased expression of *Chrng*, *Chrnb1*, *Chrnd*, *Ache*, and *Cd44* (Fig. 7E), all previously shown to increase when NMJs are stressed [40–42]. In stark contrast, we found reduced levels of *Fgf7* and *Fgfbp1 (*Fig. 7E*)*, which have been shown to play important roles in the formation, maintenance and repair of NMJs [43, 44]. However, genes encoding synaptic basal lamina proteins which are known to be enriched at the NMJ [45, 46] were unchanged in the soleus of *Megf10* KO mice (Fig. S9).

To validate our RNAseq findings, we examined levels of *Myh3*, *Pax7*, *Chrng*, and *Fgfbp1* in the soleus of WT and *Megf10* KO mice using qPCR. As displayed in Fig. 7F, qPCR showed all four genes altered and in the same direction as found using RNAseq in *Megf10* KO mice. Overall, transcriptional analysis revealed molecular mechanisms dysregulated in *Megf10* KO muscles with important roles in muscles and at the NMJ.

## Discussion

In this study, we identified cellular and molecular changes in muscles and neuromuscular junctions (NMJs) of *Megf10* knockout (KO) mice. *Megf10* KO muscles have decreased mass due to reduced numbers of muscle fibers and reduced growth of muscle fiber size. Although muscle fibers that form do not exhibit obvious signs of degeneration, their NMJs present with structural abnormalities associated with aging and diseases. These findings suggest that *MEGF10*-related myopathy is caused by impaired myogenesis and NMJ instability. In support of this possibility, molecular analyses revealed changes in levels of genes and pathways with important roles in myogenesis and at the NMJ. These findings provide important insights about potential cellular and molecular underpinnings of *MEGF10*-related myopathy.

### *Megf10* deletion impacts muscle fiber formation and growth

We identified novel aspects of muscle pathology which have not been previously examined in *Megf10* KO mice or *MEGF10*-related myopathy patients. For instance, we found a consistent ∼ 20% decrease in muscle fiber number in *Megf10* KO mice at all ages examined, suggesting that muscle fiber formation may be impaired in *Megf10* KO mice. As the loss of *Megf10* has been shown to decrease the self-renewal of satellite cells and drive their precocious differentiation [4, 6], there may not be enough myoblasts being produced during early development to form the same number of muscle fibers in *Megf10* KO mice as are formed in WT mice. Another possibility is that some muscle fibers degenerate early in development. Future studies should assess whether impaired myogenesis or degeneration of muscle fibers during the early stages of development underlie the reduced number of muscle fibers in *Megf10* KO mice. We also found that muscle fibers fail to continue to grow in *Megf10* KO mice as they transition into middle-age. This absence of muscle growth may also be linked to impaired self-renewal of satellite cells.

### Evidence that loss of *Megf10* may directly affect the NMJ

*Megf10* has been shown to localize to the NMJ [1] and to be expressed by perisynaptic Schwann cells (PSCs) [5, 31]. We discovered that loss of *Megf10* causes PSCs to extend processes into the synaptic cleft, which has been shown to adversely affect the stability of the NMJ [28, 29]. In support of this, the presynapse presents with abnormally low density of synaptic vesicles and mitochondria, postsynaptic junctional folds are diminished, and the postsynaptic nAChRs are highly fragmented in *Megf10* KO NMJs. While we did not observe NMJ denervation in *Megf10* KO mice, we did find areas of the presynapse that were not in direct apposition to the postsynapse, suggesting that *Megf10* may be important for proper alignment of the motor axon terminal. We also found altered expression of genes with important roles in NMJ maintenance and repair in *Megf10* KO mice. These data strongly suggest that NMJs are also a site of pathology in *MEGF10*-related myopathy. Based on these findings, future studies should determine the extent of NMJ degeneration in patients with *MEGF10*-related myopathy, especially since treatment development for this disease has focused solely on the role of *Megf10* in myogenesis [47].

It is also worth noting that we found NMJs progressively acquire structural abnormalities in otherwise healthy appearing muscle fibers in juvenile, young adult, and middle-aged *Megf10* KO mice. Thus, instability at NMJs may eventually compromise the health of muscle fibers. In support of this possibility, muscle fibers fail to increase in size as *Megf10* KO mice transition into middle-age in contrast to muscle fibers in control mice.

There are several ways by which the loss of *Megf10* may affect the stability of the NMJ. The loss of *Megf10* may adversely affect the NMJ by causing PSCs and motor axon terminals to acquire abnormal morphological features. However, it is possible that the effects of *Megf10* deletion on PSCs and motor axon terminals may be due to changes elsewhere in skeletal muscles and in the spinal cord. In future studies, *Megf10* should be deleted specifically from PSCs to uncover its function in these cells. Such studies may unearth additional functions of MEGF10 at the NMJ such as regulation of cellular adhesion, cellular tiling, and phagocytosis, which are all functions that MEGF10 has been shown to mediate in glial cells and neurons in the central nervous system [10–12].

### NMJ degeneration varies across muscles and may precede muscle atrophy in *Megf10* KO mice

While previous studies in human patients with *MEGF10*-related myopathy found normal nerve conduction velocity and some patients with normal compound muscle action potential upon repetitive nerve stimulation [1], it is possible that the NMJ defects are only penetrant in certain muscles. For instance, in this study we found that the diaphragm in *Megf10* KO had robust NMJ defects as early as the juvenile time point, while NMJ pathology was less severe in the extensor digitorum longus (EDL) even at the middle-aged time point.

### Mice as models of human *MEGF10*-related myopathy

Modeling *MEGF10*-related myopathy is complicated by its highly variable clinical phenotype and age of onset. These range from aggressive postnatal onset caused by null mutations to *MEGF10* to adult onset with milder muscle pathology caused by certain missense mutations to *MEGF10* [1, 3]. In common among the different types of *MEGF10*-related myopathy are reduced muscle mass, variable muscle fiber size, and disruptions to muscle fiber architecture [1–3, 26]. Young adult and middle-aged *Megf10* KO mice have lower muscle mass but do not present with altered fiber size or other muscle structure abnormalities such as minicores. However, it should be noted that our data suggest that *Megf10* KO mice may eventually experience muscle atrophy. We found that adult *Megf10* KO mice have stagnant growth in muscle fiber size and transcriptomic evidence of depleted satellite cells, altered myogenesis, and declined muscle health. These findings support using *Megf10* KO mice to discover and test therapeutics to treat humans suffering with *MEGF10*-related myopathy.

## Conclusions

Our work uncovers additional effects of *Megf10* deletion on skeletal muscles. This study shows that fewer muscle fibers are formed in *Megf10* KO mice, likely due to impaired myogenesis. It also provides strong evidence that the NMJ may be a site of pathology in *MEGF10*-related myopathy during the postnatal phase of life possibly due to aberrant changes in the motor axon terminal and in PSCs. Additionally, we uncovered genes and molecular pathways dysregulated in *Megf10* KO mice that could be targeted to treat the disease or at a minimum to test the efficacy of therapeutic interventions using mice lacking *Megf10*. Overall, this study provides cellular and molecular clues to develop treatments for *Megf10*-related myopathy.

### Electronic supplementary material

Below is the link to the electronic supplementary material.

Supplementary Material 1

## Data Availability

The data that support the RNA-seq findings of this study are available in NCBI GEO athttps://www.ncbi.nlm.nih.gov/geo, reference number GSE256199 [47]. All other supporting data are available from the corresponding author on reasonable request.

## Abbreviations

EDL: Extensor digitorum longus
FACS: Fluorescence activated cell sorting
fBTX: Fluorophore–conjugated alpha bungarotoxin
fWGA: Fluorophore–conjugated wheat germ agglutinin
nAChR: Nicotinic acetylcholine receptor
NMJ: Neuromuscular junction
PSC: Perisynaptic Schwann cell
SC: Schwann cell
TA: Tibialis anterior
VAChT: Vesicular acetylcholine transporter
