# Supplementary material for: Cellular and molecular alterations to muscles and neuromuscular synapses in a mouse model of MEGF10-related myopathy
Source: Skelet Muscle. 2024 May 17;14:10. doi: 10.1186/s13395-024-00342-6 (PMC11100254; doi:10.1186/s13395-024-00342-6)
Supplement: Supplementary file 1 — Supplementary Material 1 [file 13395_2024_342_MOESM1_ESM.pdf]

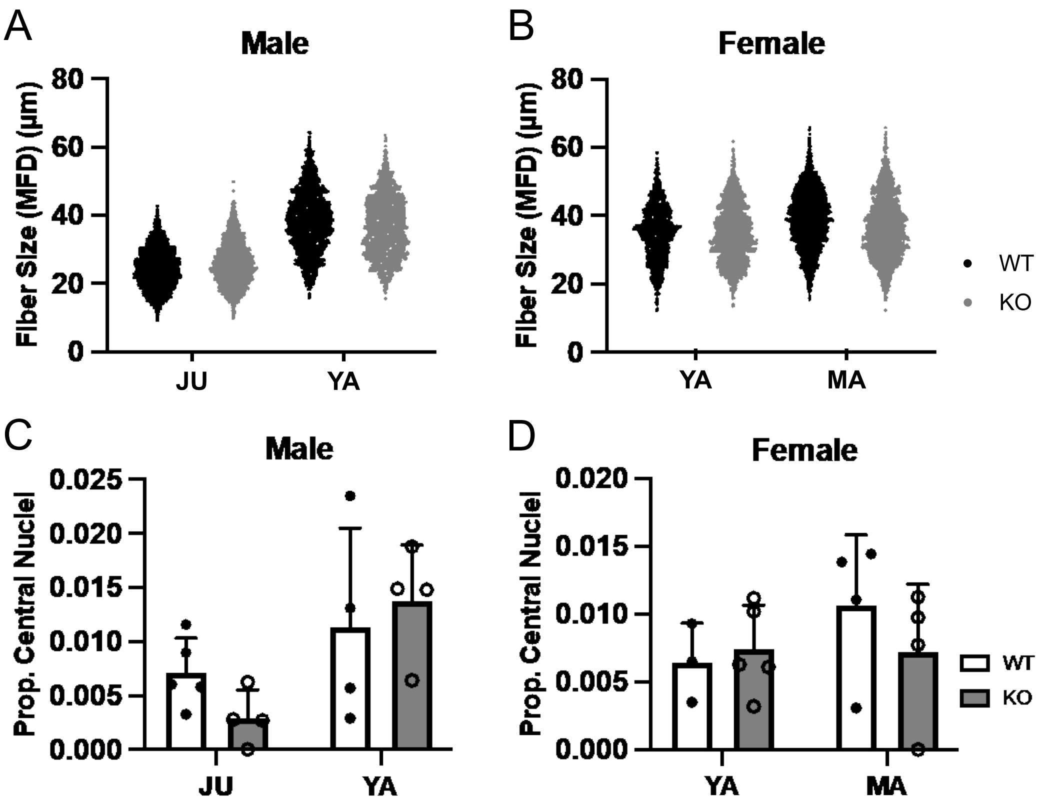

SUPPLEMENTAL FIGURE 1. Muscle fibers are not atrophying from juvenile through middle-age in *Megf10* KO mice. Tibialis anterior (TA) cross sections were examined for distribution of fiber sizes (minimum Feret diameter (MFD)) in (A) male juvenile and young adult WT and *Megf10* KO mice and (B) female young adult and middle-aged WT and *Megf1*0 KO mice. TA cross sections were also examined for the prevalence of centralized nuclei in muscle fibers in (C) male juvenile and young adult WT and *Megf10* KO mice and (D) female young adult and middle-aged WT and *Megf10* KO mice. Two-way ANOVA with Tukey's multiple comparison test for statistical analysis. Values represented as mean + SD. JU (juvenile, 1 mo), YA (young adult, 3 mo), MA (middle-aged, 12-14 mo).

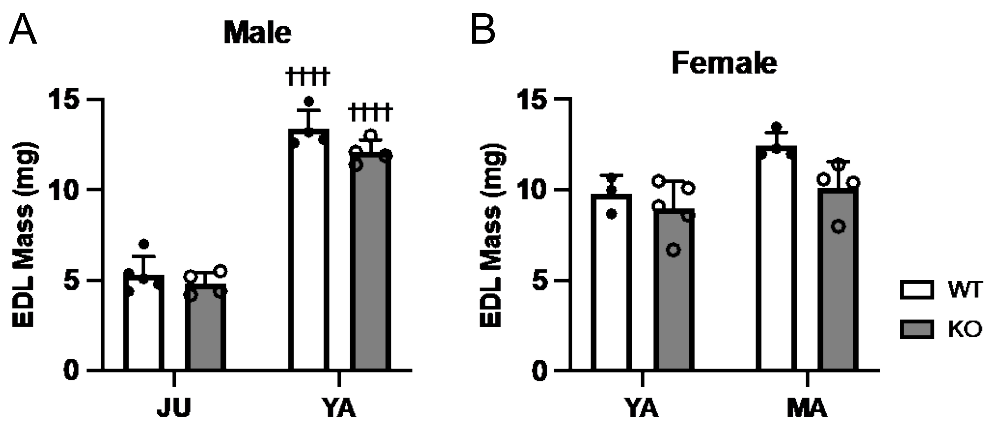

SUPPLEMENTAL FIGURE 2. EDL muscle mass is not affected in *Megf10* KO mice. Whole extensor digitorum longus (EDL) muscles were weighed from (A) male juvenile and young adult WT and *Megf10* KO mice and (B) female young adult and middle-aged WT and *Megf10* KO mice. Two-way ANOVA with Tukey's multiple comparison test for statistical analysis. Values represented as mean + SD. $^{††††}p < 0.0001$ versus juvenile. JU (juvenile, 1 mo), YA (young adult, 3 mo), MA (middle-aged, 12-14 mo).

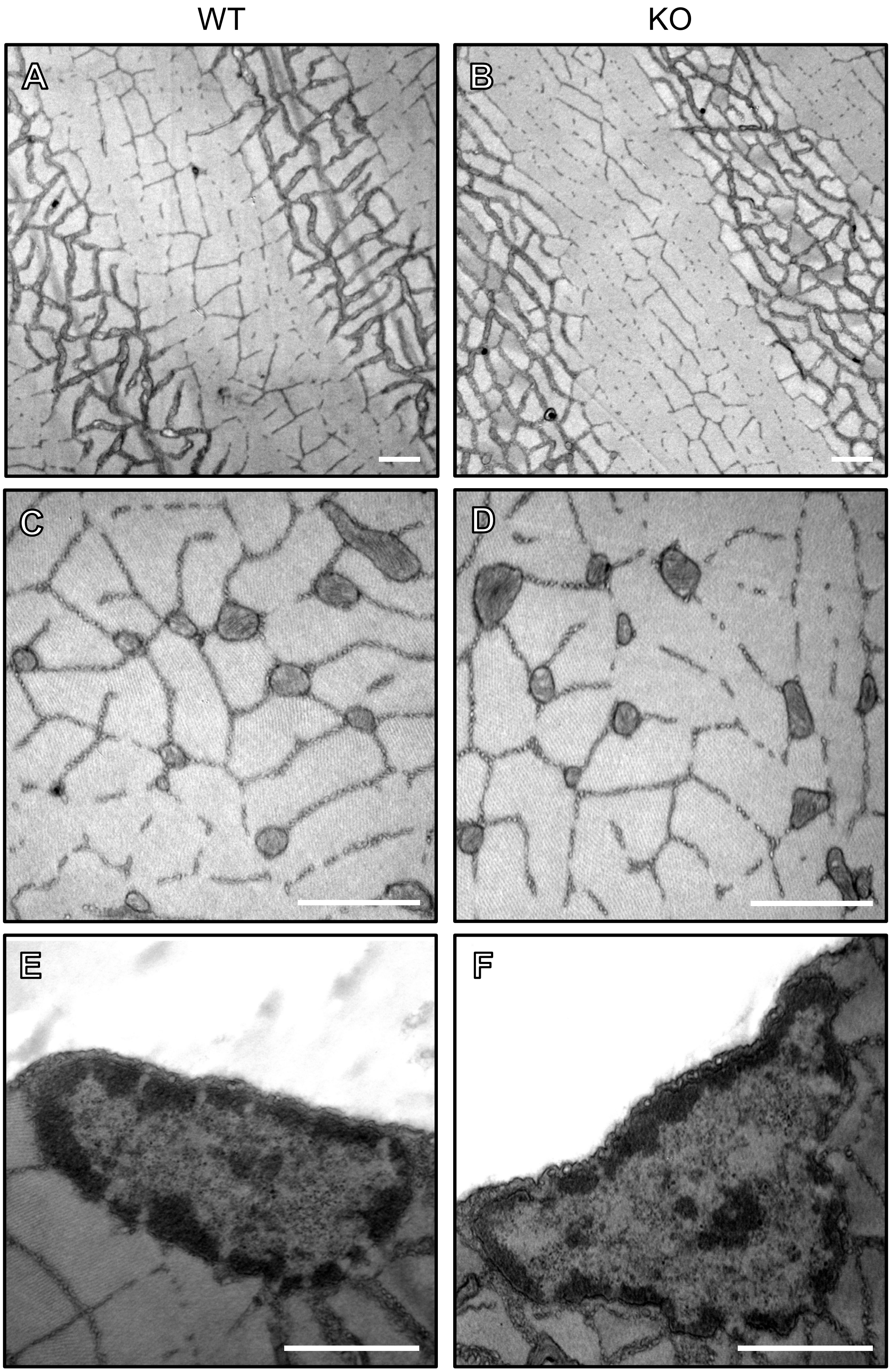

SUPPLEMENTAL FIGURE 3. Ultrastructure of muscle is unaffected by *Megf10* KO. Representative transmission electron micrographs of cross sections from young adult (6 mo) female WT and *Megf10* KO extensor digitorum longus (EDL) muscles which are (A,B) zoomed out views of muscle fibers, (C,D) zoomed in views of muscle fibers, and (E,F) zoomed in views of myonuclei. Scale bars are 1 μm.

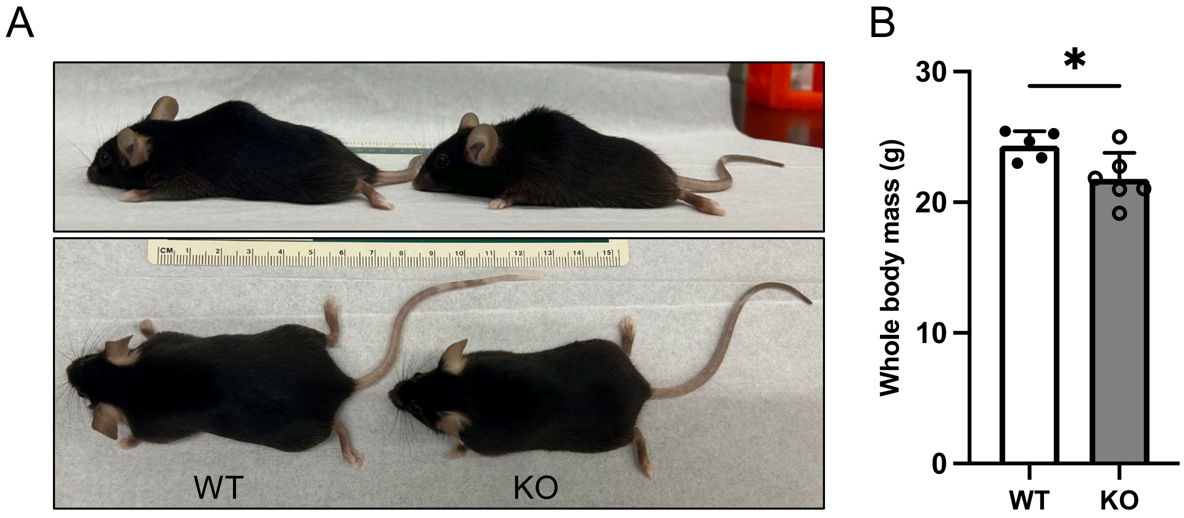

SUPPLEMENTAL FIGURE 4. Body mass is decreased in *Megf10* KO mice. (A) Representative images of middle-aged female WT and *Megf10* KO mice. The top panel (side view) shows that WT and *Megf10* KO mice have a similar degree of kyphosis, while the bottom panel (aerial view) shows that Megf10 KO mice may be shorter in length. (B) Whole body mass in female middle-aged (10-14 mo) WT and *Megf10* KO mice. Unpaired 2-sided Student's t-test for statistical analysis. Values represented as mean + SD. *$p < 0.05$ versus WT.

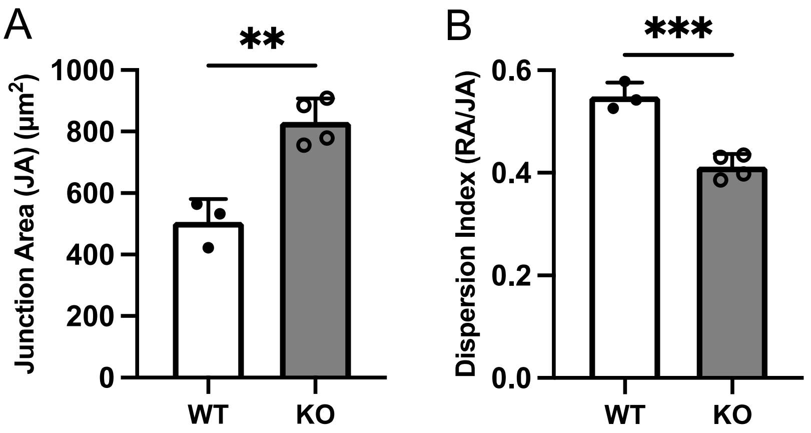

SUPPLEMENTAL FIGURE 5. NMJ junctional area and dispersion index are altered in *Megf10* KO mice. The fBTX staining in young adult (3 mo) male WT and Megf10 KO diaphragms was analyzed for (A) junctional area (area of a perimeter around the nAChRs) and (B) dispersion index (receptor area / junctional area). Unpaired 2-sided Student's t-test for statistical analysis. Values represented as mean + SD. $^{**}p < 0.01$, $^{***}p < 0.001$ versus WT.

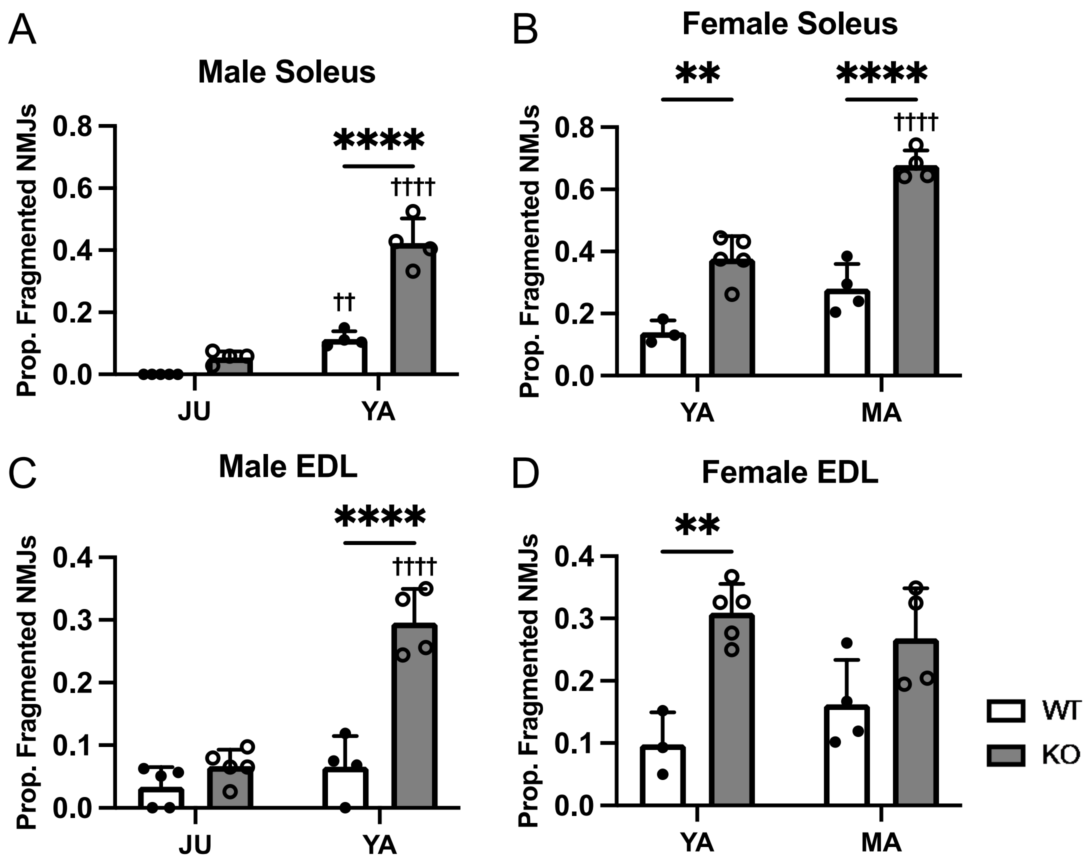

SUPPLEMENTAL FIGURE 6. NMJ postsynaptic fragmentation in the soleus and EDL of *Megf10* KO mice. The postsynaptic stain for the NMJ was analyzed for the proportion of NMJs with postsynaptic fragmentation (>4 distinct nAChR islands) in the (A-B) soleus and (C-D) extensor digitorum longus (EDL) muscles. Two-way ANOVA with Tukey's multiple comparison test for statistical analysis. Values represented as mean + SD. $^{**}p < 0.01$, $^{****}p < 0.0001$ versus WT. $^{\dagger\dagger}p < 0.01$, $^{\dagger\dagger\dagger\dagger}p < 0.0001$ versus (A,C) juvenile or (B,D) young adult. JU (juvenile, 1 mo), YA (young adult, 3 mo), MA (middle-aged, 12-14 mo).

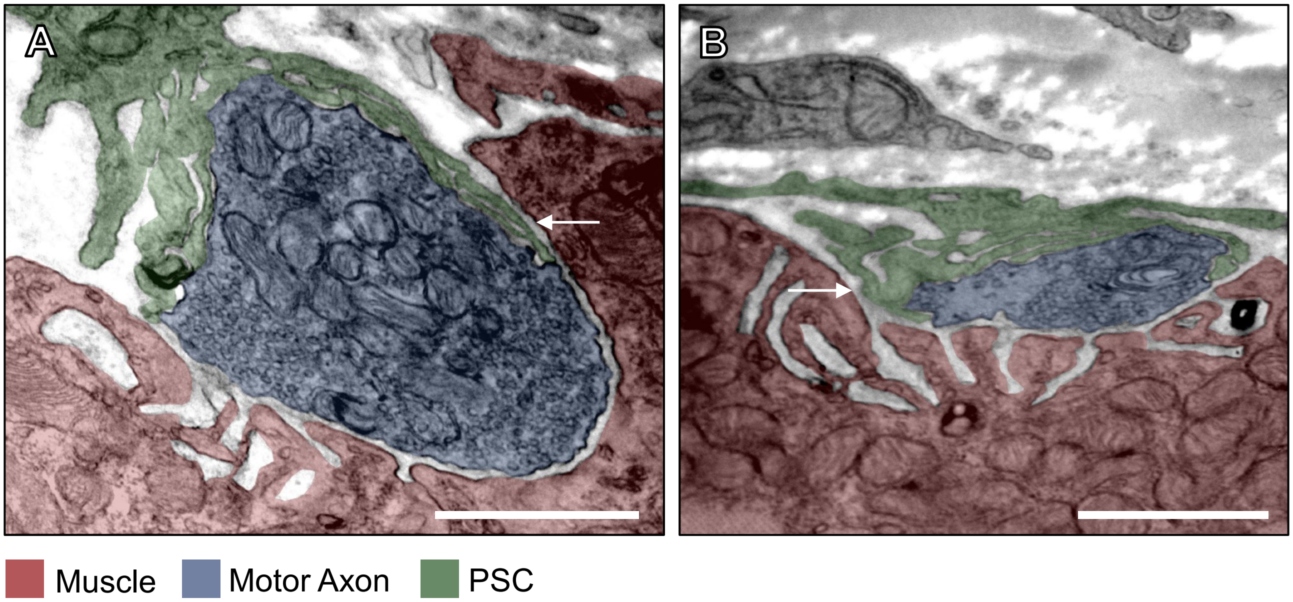

SUPPLEMENTAL FIGURE 7. PSC processes extend far into the synaptic cleft in *Megf10* KO mice. (A,B) Transmission electron micrographs of NMJs in cross sections from young adult (6 mo) female *Megf10* KO extensor digitorum longus (EDL) muscles show PSC processes extending deep into the synaptic cleft (arrows).

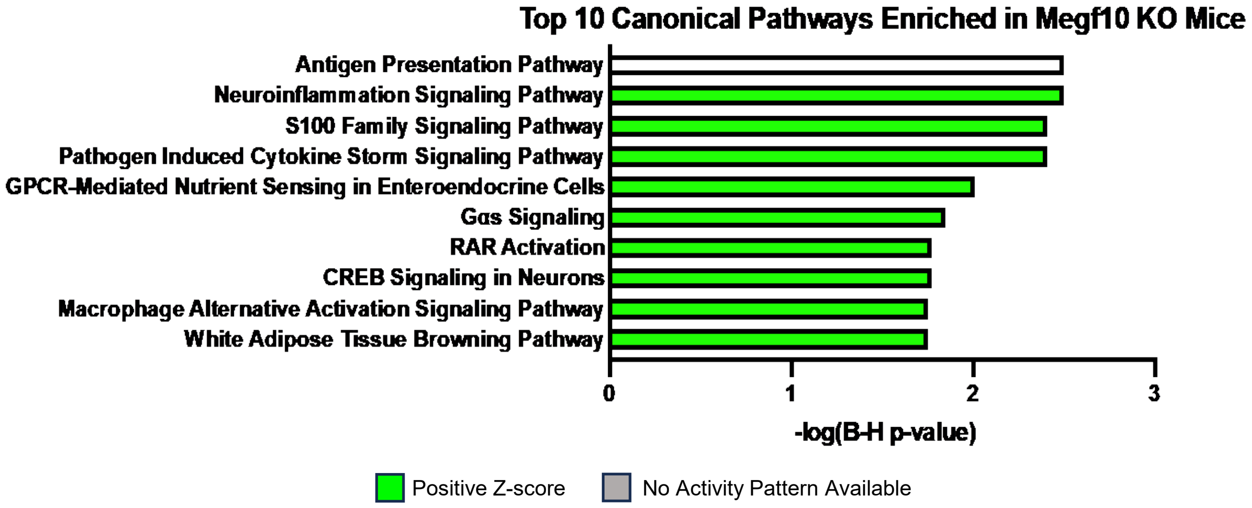

SUPPLEMENTAL FIGURE 8. Ingenuity Pathway Analysis of RNA-seq data. Ingenuity Pathway Analysis (IPA) was used to identify the top altered pathways in *Megf10* KO muscle using RNA-seq data collected from whole soleus muscles isolated from young adult (7 mo) female WT and *Megf10* KO mice (n = 4).

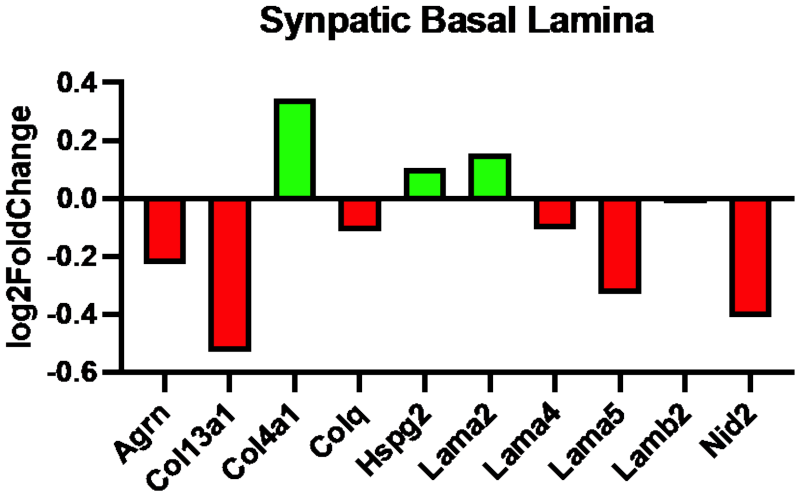

SUPPLEMENTAL FIGURE 9. Expression of genes related to the synaptic basal lamina are unchanged in *Megf10* KO muscle. Bulk RNA-seq performed on whole soleus muscles isolated from young adult (7 mo) female WT and *Megf10* KO mice (n = 4) examined the expression of components of the synaptic basal lamina which have been shown to be enriched in the NMJ synaptic cleft and/or play an important role in junctional fold formation. Fold changes are displayed as relative expression in *Megf10* KO muscle compared to WT muscle.
